# Study of Hydatidosis-Attributed Mortality in Endemic Area

**DOI:** 10.1371/journal.pone.0091342

**Published:** 2014-03-14

**Authors:** Moncef Belhassen-García, Angela Romero-Alegria, Virginia Velasco-Tirado, Montserrat Alonso-Sardón, Amparo Lopez-Bernus, Lucia Alvela-Suarez, Luis Perez del Villar, Adela Carpio-Perez, Inmaculada Galindo-Perez, Miguel Cordero-Sanchez, Javier Pardo-Lledias

**Affiliations:** 1 Seccion de Enfermedades Infecciosas, Servicio de Medicina Interna, Complejo Asistencial Universitario de Salamanca, CIETUS, IBSAL, Salamanca, Spain; 2 Servicio de Medicina Interna, Complejo Asistencial Universitario de Salamanca, IBSAL, Salamanca, Spain; 3 Servicio de Medicina Interna, Complejo Asistencial Universitario de Salamanca, CIETUS, IBSAL, Salamanca, Spain; 4 Departmento de Medicina Preventiva, Salud Publica y Microbiologia Medica, Universidad de Salamanca, Salamanca, Spain; 5 CIETUS, IBSAL, Universidad de Salamanca, Salamanca, Spain; 6 CAP Camargo, José Barros, Cantabria, Spain; 7 Servicio de Medicina Interna, Hospital General de Palencia “Río Carrión”, Palencia, Spain; Oxford University, United Kingdom

## Abstract

**Background:**

Cystic hydatid disease is still an important health problem in European Mediterranean areas. In spite of being traditionally considered as a “benign” pathology, cystic echinococcosis is an important cause of morbidity in these areas. Nevertheless, there are few analyses of mortality attributed to human hydatidosis.

**Objective:**

To describe the epidemiology, the mortality rate and the causes of mortality due to *E. granulosus* infection in an endemic area.

**Methodology:**

A retrospective study followed up over a period of 14 years (1998–2011).

**Principal Findings:**

Of the 567 patients diagnosed with hydatid disease over the period 1998–2011, eleven deaths directly related to hydatid disease complications were recorded. Ten patients (90.9%) died due to infectious complications and the remaining one (9.1%) died due to mechanical complications after a massive hemoptysis. We registered a case fatality rate of 1.94% and a mortality rate of 3.1 per 100.000 inhabitants.

**Conclusions:**

Hydatidosis is still a frequent parasitic disease that causes a considerable mortality. The main causes of mortality in patients with hydatidosis are complications related to the rupture of CE cysts with supurative collangitis. Therefore, an expectant management can be dangerous and it must be only employed in well-selected patients.

## Introduction

Human cystic echinococcosis (CE) is a zoonotic infection caused by *Echinococcus granulosus*, and it causes above 1.009 DALYs (disability-adjusted life year) and annual cost of US 763 million dollars worldwide, brining its socioeconomic impact higher than Chagas or Hansen's disease [1]. Furthermore, there are areas where this infection is highly endemic, such as South America, China, Africa, and European Mediterranean countries. A survey performed in the region of Salamanca, Castilla y Leon (located in the northwest of Spain) estimated the incidence of hydatid disease as 12 per 100.000 inhabitants per year during the period of 1996–2003. Moreover, the seroprevalence in the same region was above 2% [2], [3]. In addition, we detected autochthonous pediatric patients, a clear sign of active local transmission of disease [4].

Cystic echinococcosis (CE) is usually asymptomatic and it has traditionally been considered a “benign” pathology. However, CE occasionally results in a complicated cyst. Rupture of the hydatid cyst and compression of pericystic structures are the most frequent complications related to CE. Therefore, depending on the hydatid cystic localization, CE may cause bile duct obstruction, pleural fistula or other disorders [5]. In addition, hypersensitivity reactions caused by circulating immune complexes and the activation of complement pathway which give rise to glomerulonephritis and anaphylactoid reactions are other complications frequently associated with hydatidosis disease [6], [7]. Furthermore, we recently reported that the CE can become frequently super-infected by other microorganisms such as bacterial and fungal infections [8]. Thus, despite the available therapeutic alternatives, there is still a certain mortality rate directly related to hydatid cystic complications [9]. The aim of the present study is to describe the mortality epidemiology, the clinical settings and the treatment applied to patients who died due to hydatidosis our area.

## Materials and Methods

The design was an observational retrospective study. We reviewed all patients diagnosed with CE admitted between January 1998 and December 2011 at the University Hospital of Salamanca, a tertiary care hospital for a population of 350.000 inhabitants located in western Spain. Diagnosis of CE was considered in the following circumstances: *i*) direct parasitological diagnosis *ii*) diagnosis obtained by radiological methods (ultrasonography and/or computerized axial tomography) or serology. Next, we selected all patients whose deaths are due to complications of hydatidosis (infectious, mechanical, allergic or other complications). Patients whose deaths were not related to hydatidosis were excluded from the analysis.

We calculated the cumulative incidence (it measures the risk of an event happening), and the incidence rate (number of new cases per unit of population and time). In addition, we measured the case fatality rate, or proportion of people who contract a disease and die as its consequence in a specific area during an established period of time. The case fatality rate is considered as a marker of severity or virulence representing the risk of death of those affected by a certain disease over a period of time. We also calculated the mortality rate, which marks the proportion of deaths due to a certain disease during a period of time in a population. The descriptive results are expressed as means, SDs, and percentages. We further analyzed the association between mortality and other variables using Odds Ratio (OR) with its 95% confidence interval (95% C.I.). All p-values reported for OR were calculated using Fisher's exact test. The *p* value of significance on Fisher's exact test was set at *p*<0.05. Statistical analyses were carried out using the SPSS Statistical Package (SPSS Inc., Chicago, IL).

### Ethics statements

This study was approved by the Ethics Committee of Complejo Universitario Asistencial de Salamanca (CAUSA). All data analyzed were anonymized. As it is a mortality study, written consent was not obtained and it was specifically waived by the approving IRB.

## Results

During the period 1998–2011, 567 patients were diagnosed with hydatidosis in the healthcare area of Salamanca. It represents a cumulative incidence of 1.62 cases per 1.000 people and an incidence rate of 11.52 cases per 100,000 inhabitants every year. The demographic and clinical characteristics are outlined in the table 1.

**Table 1:** Demographic characteristics of our cohort of patients with hydatidosis.

Variable	n 567
**Age mean±SD (years)**	59.7±20.1
**Sex male (%)**	320 (56.4)
**Location (%)**	
Liver	414 (73.1)
Lung	72 (12.8)
Liver & Lung	18 (3.2)
Other	62 (10.9)
**Treatment specific (%)**	
Surgery	342 (60.3)
PAIR*	0
Antiparasitic drug	223 (39.4)
Wait and see	225 (39.7)

*PAIR: Puncture, Aspiration, Injection, and Reaspiration.

Of these patients, 32 (5.6%) died: 21 (65.6%) of these cases were not associated to CE mainly heart failure 5 (15.6%), cancer 4 (12.5%), infection unrelated 2 (6.3%), chronic obstruction pulmonary disease (1, 3.1%) or other meanwhile 11 (34.3%) patients died as a direct cause of hydatid disease or its complications (table 2). These data represents a case fatality rate of 1.94% and a mortality rate of 3.1 per 100,000 people. The main features of the patients who died of hydatidosis are shown in table 3. These patients had an average age of 79.2±9.1 years. Four patients were female; six patients had any type of immunodepression (diabetes mellitus, steroid therapy or other) and the average number of chronic diseasses such as heart failure, chronic obstructive pulmonary disease (COPD), cancer, chronic kidney failure, diabetes mellitus and other was 2.3±1.2.

**Table 2:** Cause of death of patients with cyst echinococcosis (CE).

Variable	n 567
**Complication CE (%)** *	11 (34.3)
Biliary fistula	4 (12.5)
Portal hypertension	2 (6.3)
Peritonitis	1 (3.1)
Superinfection	1 (3.1)
Compression of spinal cord	1 (3.1)
Infection of surgical wound	1 (3.1)
Massive hemoptisis	1 (3.1)
**Heart Failure**	5 (15.6)
**Cancer**	4 (12.5)
**Other infections**	2 (6.3)
**Chronic obstruction pulmonary disease**	1 (3.1)
**Other**	7 (21.9)
**No data**	2 (6.3)
**Total**	32 (100)

*Contribution of each one of these causes in the mortality global: number and percentage of total mortality.

**Table 3:** Main characteristics of the patients who died from hydatidosis.

N	Sex	Age (years)	Diagnosis (month)	Location (WHO)	Specific treatment*	Reason for admission	Complications	Treatment	Microorganisme	Cause of death
1.	F	80	0,2	Lung & Liver (Grade IV)	Albendazole	Sepsis colangitis	Fistula hepatobronchial	Percutaneous drainage Antibiotic	Negative	Septic shock secondary to colangitis
2.	M	95	12	Liver (Grade III)	Not	Sepsis Colangitis	Biliary obstruction	Surgery Antibiotic	Unrealized	Septic shock secondary to colangitis
3.	F	85	1	Liver (Grade III-IV)	Albendazole	Sepsis Colangitis	Biliary obstruction	Percutaneous drainage Antibiotic	Negative	Septic shock secondary to colangitis
4.	M	73	1	Liver (Grade II)	Not	Sepsis Colangitis	Biliary obstruction	Surgery Antibiotic	Escherichia coli	Septic shock secondary to colangitis
5.	F	87	36	Liver (Grade III-IV)	Not	Sepsis Peritonitis	Not	Antibiotic	Staphylococcus epidermidis	Septic shock secondary to bacterial peritonitis
6.	M	62	108	Liver (Grade III)	Not	Digestive bleeding	Bacterial peritonitis	Endoscopy Antibiotic	Streptococcus sp	Septic shock secondary to bacterial peritonitis
7.	M	80	2	Liver (Grade III)	Albendazole	Bacterial peritonitis	Biliary obstruction	Antibiotic	Staphylococcus warneri Pseudomonas aeruginosa	Septic shock secondary to bacterial peritonitis
8.	F	87	60	Liver (Grade II)	AlbendazolePrazicuantel	Abdominal sepsis	Superinfection	Antibiotic	Negative	Abdominal septic shock
9.	M	74	60	Spine & Lung	AlbendazolePrazicuantel	Urinary sepsis	Medular compression	Surgery Antibiotic	Escherichia coli	Septic shock secondary to urinary infection
10.	M	78	1	Liver (Grade III)	Not	Sepsis colangitis Jaundice	Biliary obstruction	ERCP♯ Surgery Antibiotic	Klebsiella oxytoca	Septic shock secondary to infection of surgical wound
11.	M	71	384	Lung & Liver (Grade II)	Not	Hemoptysis Massive	Rupture arterial bronchial	Pulmonary artery embolization Antibiotic	Unrealized	Massive hemoptysis

*Previous specific treatment before complication of CE.

♯ERCP: Endoscopic Retrograde Cholangio-Pancreatography.

The most common location of the hydatid cyst was the liver (8 cases) followed by simultaneous location of liver and lung (2 cases), and simultaneous location of lung and spine (1 case). According to the WHO classification of hepatic cyst, we reported the following CE staging: six cases had type CE3, three cases had type CE2 and one patient had type CE4. Overall survival from the time of diagnosis was 60.4±11.3 months. However, in four cases (36.3%), the time from the diagnosis to the date of death was less than one month. One patient was diagnosed more than 30 years before the date of death. Five of 11 cases (45.4%) have not undergone any specific treatment for hydatidosis before death.

With regard to hydatid cyst complications, we found that the main complications were the compression of vital structures and the rupture of hydatid cyst, which caused bile duct obstruction and supurative collangitis (4 cases). Interestingly, six patients (54.5%) presented other microbial positive culture, the most frequently identified being Escherichia coli 2(16.6%), Streptococcus spp. 1 (8.3%), Staphylococcus warneri 1(8.3%), Staphylococcus epidermidis 1 (8.3%), Pseudomonas aeruginosa 1(8.3%) and Klebsiella oxytoca 1 (8.3%). For these patients medical treatment with broad-spectrum antibiotics was the main approach used. Four patients (36.3%) were treated with surgery; two (18.2%) underwent percutaneous drainage due to their poor clinical status. Two patients died by complication of portal hypertension and cirrhosis caused by CE (excluding other causes); both of them presented bacterial peritonitis. Furthermore, both patients had grade C of Child-Pugh classification (score of prognosis of chronic liver disease). Remarkably, only one patient (9.1%) died by massive hemoptysis with a pulmonary cyst. Finally, no patient died of complications in elective surgery or CE-related allergic reactions.

Regarding the risk factors associated with mortality, the only variable associated with mortality was the age (≥65 years) OR = 9.85 [1.25–77.52], p = 0.013. Others variables such as gender OR = 1.24 [0.37–4.12], location of cyst OR = 3.2 [0.66–15.41], and specific treatment for CE OR = 1.24[0.37–4.12] were not associated with mortality.

## Discussion

It is well-known that patients diagnosed with CE may present complications that represent an important cause of morbidity. However, there are very few published studies focused on mortality of hydatidosis. Thus, the main objective of this work was to describe the mortality of CE and its causes in our area. First, we reviewed the medical records of in-patients during a 14-year period and we find a still high incidence in our area as we have previously reported in previous studies [2], [3]. Of the 567 patients included in our study, 32 in-patients (5.6%) died. Strikingly, in above one third of these patients, death occurred as a direct cause of complications related to hydatidosis. This fact shows that the complications related to CE are the main cause of mortality in these patients.

The case fatality rate and the mortality rate estimated in our work were 1.94% and 3.1 per 100,000 inhabitants per year, respectively. However these results might be understimated since only in-patients were considered. Thus, a moderate selection bias was assumed in the present study. Nevertheless, the case fatality rate repoted in our study was higher than the case fatality rate described in previous studies performed in Chile, whose numbers vary from 0.13 to 0.20 per 100,000 people year [10]. Regarding the demographics and clinical variables associated with the mortality, we report that the age was the only variable associated with mortality, being higher in patients older than 65 year. Meanwhile, there were no significant associations between mortality and other variables such as gender, location of the cyst or specific treatment. These results are in line with a previous report performed from the USA, where the highest mortality rate was found in elderly, although we did not find differences between genders [9]. Six of 11 patients died before any specific treatment for hydatidosis was undertaken because they were elderly and they had comorbidity. This data suggest that a “wait and see” approach can be dangerous and must be employed only for well selected patients.

With respect to the lethal complications related to CE. We found that the rupture of CE in the biliary tract with suppurative cholangitis was the main cause of mortality. In addition, this complication was frequently associated with co-infections by gram-positive and gram-negative bacteria. Portal hypertensión and cavernomatosis have also been found in patients with CE mainly due to compresion, invasion or portal/suprahepatic thrombosis (formally named Bud Chiari syndrome) [11], [12]. However, it should be noticed that portal hypertension can also be caused by secondary cholangitis sclerosing or secondary biliary cirrhosis after surgical treatment or use of parasiticides [13]. We found two patiens who died by complications related to portal hypertensión and cirrhosis such as bacterial peritonitis; both patients presented multiple and giant hydatid cysts (>15 cm). In this sense, we have previously reported that superinfection is one of the most important complicatons of CE in our area. Gram positive/ negative bacterial and Aspergillus species are the most frequent microbial pathogenes involved in liver and lung complications, respectively [8]. Another patient presented massive hemoptisis secondary to complicated CE. It is possible that this last patient could be also affected by aspergillus species [14], but this fact was not demonstrated.

The anaphylactoid reactions in hydatidosis may occur when the CE is broken and its fluid released spontaneously after a trauma or surgery. This reaction is usually abrupt and it can arise firstly as an anaphylactic shock and finally may cause the death of the patient [15]. In our work we did not detect any patient who died by this cause. An early diagnosis and a protocoled perioperative prophylaxis in our hospital have influenced these results [16]. Finally it is important to outline the null mortality caused by elective surgery of non complicated hydatid cyst. These results agree with other published studies which describe a low mortality rate before the surgical intervention of thoracic and liver CE [17], [18].

We conclude that hydatidosis in Spain is still a frequent parasitic disease that causes a considerable mortality higher than previously reported. The main causes of mortality in patients with hydatidosis are complications related to the rupture of CE cysts with supurative collangitis. Therefore, an expectant management can be dangerous and it must be only employed in well sellected patients.
